# Asymmetry of peripheral vascular biomarkers in ischemic stroke patients, assessed using NIRS

**DOI:** 10.1117/1.JBO.25.6.065001

**Published:** 2020-06-19

**Authors:** Yingwei Li, Yunfei Ma, Shaoqing Ma, Zhenhu Liang, Fang Xu, Yunjie Tong, Blaise deB Frederick, Shimin Yin, Xiaoli Li

**Affiliations:** a Yanshan University, School of Information Science and Engineering, Qinhuangdao, China; b McLean Hospital, McLean Imaging Center, Belmont, Massachusetts, United States; c PLA Rocket Force Characteristic Medical Center, Department of Neurology, Beijing, China; d Purdue University, Weldon School of Biomedical Engineering, West Lafayette, Indiana, United States; e Harvard Medical School, Department of Psychiatry, Boston, Massachusetts, United States; f Beijing Normal University, Center for Collaboration and Innovation in Brain and Learning Sciences, Beijing, China

**Keywords:** near-infrared spectroscopy, peripheral artery disease, resting state, systemic low-frequency oscillations, stroke

## Abstract

**Significance:** Low-frequency oscillations (LFOs) ranging from 0.01 to 0.15 Hz are common in functional imaging studies. Some of these LFOs are non-neuronal and are correlated with autonomic physiological processes.

**Aim:** We investigate the relationships between systemic low-frequency oscillations (sLFOs) measured at different peripheral sites during resting states in ischemic stroke patients.

**Approach:** Twenty-seven ischemic stroke patients (ages 44 to 90; 20 male and 7 female) were recruited for the study. During the experiments, fluctuations in oxyhemoglobin concentration were measured in the left and right toes, fingertips, and earlobes using a multichannel near-infrared spectroscopy instrument. We applied cross-correlation and frequency component analyses on the sLFO data.

**Results:** The results showed that embolization broke the symmetry of the sLFO transmission and that the damage was not limited to the local area but spread throughout the body. Among six peripheral sites, the power spectrum width of the earlobes was significantly larger than that of the fingers and toes. This indicates that the earlobes may contain more physiological information. Finally, the results of fuzzy clustering verified that sLFOs can serve as perfusion biomarkers to differentiate stroke from healthy subjects.

**Conclusions:** The high correlation values and corresponding delays in sLFOs support the hypothesis that (1) the correlation characteristics of sLFOs in stroke patients are different from those of healthy subjects. These characteristics can reflect patient condition, to an extent. Embolization in ischemic stroke patients breaks the symmetry of the body’s sLFO transmission, disrupting the balance of blood circulation. (2) sLFOs can be used as perfusion biomarkers to differentiate ischemic stroke patients from healthy subjects. Studying these signals can explicate the overall feedback/influence of pericentral interactions. Finally, peripheral sLFOs have been shown to be an effective and accurate tool for assessing peripheral blood circulation and vascular integrity in ischemic stroke patients.

## 1 Introduction

Ischemic stroke is induced by a blockage in a cerebral vessel. Approximately 795,000 people suffer ischemic strokes every year in the United States.^1^ Emergency reperfusion therapy can restore perfusion and mitigate cognitive dysfunction.^2^ However, this treatment leads to increased risk of cerebral hemorrhage and cerebral hernia.^3^ Therefore, there is an urgent need for effective vascular perfusion biomarkers that can identify patients most likely to benefit from reperfusion therapy.

Low-frequency oscillations (LFOs) are spontaneous variations in hemodynamic parameters common in functional near-infrared spectroscopy studies, in which they are often interpreted as indicative of neuronal activity.^4^^–^^8^ However, some of these LFOs are non-neuronal. These LFOs have been found to “move” with the blood throughout the body. The origin of these oscillations is unclear, and the terminology describing them (e.g., spontaneous oscillations, low frequency waves, and v-signals) is confusing.^9^^–^^12^ Recent reviews^13^^–^^15^ in the field of physiology have pointed out that they refer to different concepts. To differentiate them from the neural LFOs, we refer to these physiological fluctuations (∼0.1  Hz) as systemic LFOs (sLFOs). They are important biomarkers that carry physiological information and are especially useful for detecting and monitoring circulatory dysfunction.^16^

In previous simultaneous fMRI/NIRS studies,^17^ resting state fMRI data have been obtained from healthy subjects, while LFO signals (i.e., oxyhemoglobin) have been recorded simultaneously with NIRS in the subjects’ fingers and toes. LFO [measured by near-infrared spectroscopy (NIRS)] in the peripheral sites and LFO (measured by BOLD-fMRI) in the brain have been shown to be correlated with delays of up to several seconds. This indicates that a portion of the LFO carries information about circulation throughout the entire body.^16^ Our previous studies^18^ of healthy subjects have shown that the sLFO signals in the symmetric peripheral sites correlate with time delays close to zero, whereas the correlation coefficients decrease between the sLFO signals of asymmetric sites, with delays of up to several seconds. This is consistent with the hypothesis that these sLFOs originate in the cardiovascular system and travel throughout the body via the bloodstream. Due to the shorter vascular path to the earlobes, the sLFOs reach the earlobes first, followed by the fingertips, and then the toes.^18^ The present study establishes baseline measures for developing perfusion biomarkers to assess peripheral vascular integrity in healthy subjects.^18^

These observations indicate that sLFOs are suitable for ischemic stroke research for the following reasons. First, embolization in the blood vessels of ischemic stroke patients changes their blood transmission pattern; sLFOs can be treated as natural “tags” on the flow of blood that record changes in blood transmission characteristics. These propagation characteristics can be extracted by investigating the sLFOs’ correlation and time delay in the periphery to evaluate the vascular integrity of ischemic stroke patients. Second, embolism in ischemic stroke patients may be present in multiple vessels. Thus, sLFO detection in the earlobes, fingers, and toes is beneficial to systemic study. Finally, these peripheral positions have simple tissue structures, leading to signal-to-noise (SNR) that is superior to functional brain studies in which light must penetrate multiple layers of scattering media (i.e., the skin and skull) before reaching the cortex.

In this study, we investigate the asymmetry of these peripheral vascular biomarkers in ischemic stroke patients. This study had two goals: (1) determine the correlation and time delay between sLFOs’ symmetric and asymmetric positions in ischemic stroke patients. These results could be used to assess vascular integrity in ischemic stroke patients; and (2) determine the effect of embolization on sLFO transmission in the human body and how these sLFOs “propagate” to the other parts of the periphery, thus, demonstrating that sLFOs can serve as perfusion biomarkers to differentiate ischemic stroke patients from healthy subjects. Finally, we use a fuzzy *c*-means clustering algorithm to verify our speculation.

## 2 Materials and Methods

### 2.1 Multichannel Research Oximeter

**Fig. 1:**
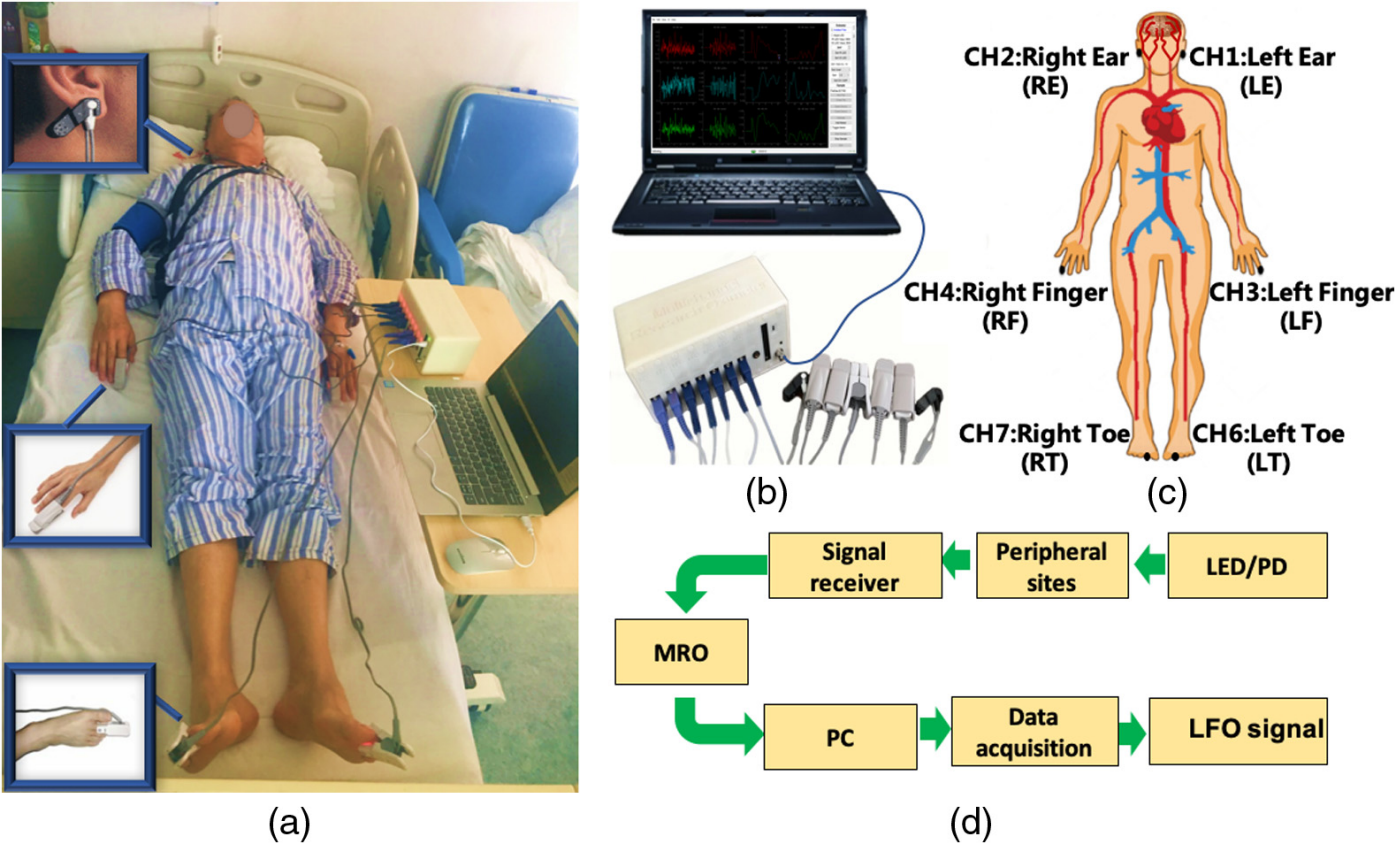
(a) The data acquisition process using multichannel oxygen monitor on ischemic stroke patients in resting states. (b) A serial port line connected MRO and PC, and (c) the subjects’ peripheral sites were LE, RE, LF, RF, LT, and RT. (d) The process of sLFOs signal extraction.

Standard commercial plethysmographs (i.e., pulse oximeters) were optimized to calculate pulse frequency and oxygen saturation. This typically means that they measure only a single channel and remove low frequency signals with a high-pass filter to focus on the cardiac waveform. To record sLFOs at multiple peripheral positions, an improved device based on standard photoplethysmograph was needed. By improving the device’s hardware and software, the sLFOs can be measured through a multichannel NIRS oximeter [multichannel research oximeter (MRO)].^19^ The device is shown in Fig. 1. It consists of seven NIRS channels and one electrocardiography channel. The core of each NIRS channel is an Olimex MOD-PULSE oximeter development board (Olimex, Ltd., Plovdiva Bulgaria). The device’s sampling frequency is 31.25 Hz. Light with wavelengths of 660 and 920 nm was used in this study.^20^^,^^21^ More information about the device can be found in previous literature.^19^ In the current experiments, measurements were performed at six positions: left earlobe (LE), right earlobe (RE), left index finger (LF), right index finger (RF), left index toe (LT), and right index toe (RT).

### 2.2 Protocols

Twenty-seven ischemic stroke subjects (7F, 20M, age=70.1±11.4  y.o.; mean±SD, range=44 to 90 y.o.) participated in this study. The institutional review committee at Rocket Force Characteristic Medical Center approved the protocol, and all subjects received a detailed explanation of the measurements to be performed. They also provided informed consent before participating in the experiments. Six pulse oximeter probes were placed at the peripheral measurement positions, as shown in Fig. 1, LE, RE, LF, RF, LT, and RT. Data were then simultaneously recorded for all channels using the MRO. The subjects were in a supine position on a comfortable bed, with their eyes open, in a room with a comfortable temperature. They were instructed to relax and breathe evenly. The subjects were then asked to remain still in a resting-state during the experiments. An MRO sampling frequency of 31.25 Hz was high enough to sample sLFOs and cardiac information without aliasing.

### 2.3 Data Analysis Methods

Analyses were computed using MATLAB (The MathWorks, Inc.). Measured data from peripheral positions at the two wavelengths (660 and 920 nm) were converted to the product of hemoglobin concentration changes for Δ[HbO] and Δ[HbR]. This followed the modified Beer–Lambert equation. ^22^^,^^23^ For the differential path length factors, we used published values of 6.51 at 660 nm and 5.86 at 920 nm. In this study, ischemic stroke patients had diminished limb control due to cerebral ischemia. This led to tremor or uncontrolled limb movement during the experiment. This resulted in sudden changes in light intensity, which are referred to as motion artifacts. Two kinds of motion artifacts were observed in the data: (1) unexpected sudden movements (e.g., sweeping gestures and sudden head movements) were a major source of “spikes” in the signal, but these motion artifacts were rare and (2) spontaneous quivers (i.e., stroke patients’ neurological disorders due to vascular thrombosis). Two methods were applied to reduce the impact of motion artifacts: (1) cubic-spline interpolation^24^ and (2) kurtosis-based wavelet filter.^25^ The low-frequency component of Δ[HbO] and Δ[HbR] was bandpass filtered (0.01 to 0.15 Hz; zero-phase Butterworth).^26^ In this study, the analyses focused on Δ[HbO], which has a higher SNR ratio than that of ΔHb.^16^^,^^27^^,^^28^

After removing the unstable signals at the beginning and end of data collection, we had 600s data from 25 patients and 480s data from two patients. To increase the sample set’s fault tolerance, we processed data through a moving window and divided the 600s data into 11 subsets: 0 to 300s, 30 to 330s, 60 to 360s, 90 to 390s, 120 to 420s, 150 to 450s, 180 to 480s, 210 to 510s, 240 to 540s, 270 to 570s, and 300 to 600s. We divided the 480s data into seven subsets: 0 to 300s, 30 to 330s, 60 to 360s, 90 to 390s, 120 to 420s, 150 to 450s, and 180 to 480s. This allowed us to assess the time delays’ stability and calculate the correlation coefficients over the entire data set. The time delays and correlation coefficients were calculated with MATLAB (i.e., xcorr). We restricted the search range for time delays to ±20s, according to the global circulation time.^29^ We used thermodynamic diagrams to explore the time delay characteristics and correlation coefficients in various types of ischemic stroke patients. To understand the power spectral component of sLFOs in ischemic stroke patients, we calculated the sLFOs’ spectral density for different types of ischemic stroke and the average power spectral density of all subjects. To explore the differences between the sLFO transmission in ischemic stroke patients and healthy subjects, we used the rank-sum test in MATLAB (i.e., ranksum) to extract the characteristic values of significant differences.

To verify that sLFOs can serve as perfusion biomarkers to differentiate ischemic stroke and healthy subjects, we needed to cluster the sLFOs’ significance difference values. There are only 27 cases of ischemic stroke patients in this study; this sample dataset is too small and thus unsuitable for supervised learning algorithms. Such a small sample will lead to an unreasonable test formula. Furthermore, the curve relationship between independent variables (time delays and correlation coefficients) and dependent variables (ischemic stroke patients and healthy subjects) is uncertain. According to these characteristics, a fuzzy *c*-means clustering algorithm is more suitable for this study. FCM clustering is an unsupervised technique that has been applied to clustering, feature analysis, and classifier designs in the fields of geology, target recognition, astronomy, medical imaging, and image segmentation. It is an important data processing tool for clustering objects.^30^^,^^31^ In this study, FCM achieved the aim of classification by optimizing the objective function and obtaining the membership degree of each sample point to all class centers. This determines the classification of sample points. The clustering loss function of the membership equation is

$$J_{f}=\overset{k}{\underset{j=1}{\sum }}\overset{n}{\underset{i=1}{\sum }}{\left[\mu_{j}(\chi_{i})\right]}^{b}{\left\|\chi_{i}-m_{j}\right\|}^{2},\;1\leq b<\infty .$$ (1)

$\overset{k}{\underset{j=1}{\sum }}\mu_{j}(x_{i})=1b$ is the weighted index, also known as the smoothing index. There is no theoretical support for the value of b, which is 2 in most cases. In a given aggregate of the data $X=(x_{1},x_{2},\cdots \cdots x_{n})$, k is the number of categories. $m_{j}$ (j=1,2,3,⋯⋯,k) is the center of each cluster. $\mu_{j}(x_{i})$ is the membership function of the i’th sample corresponding to the j’th class.

The necessary conditions for the minimum in Eq. (1) are obtained by setting the partial derivative of $J_{f}$ to zero, with respect to $m_{j}$ and $\mu_{j}(x_{i})$:

$$m_{j}=\frac{\overset{n}{\underset{i=1}{\sum }}{\left[\mu_{j}(\chi_{i})\right]}^{b}\chi_{i}}{\overset{n}{\underset{i=1}{\sum }}{\left[\mu_{j}(\chi_{i})\right]}^{b}},$$ (2)

$$\mu_{j}(\chi_{i})=\frac{{\left\|\chi_{i}-m_{j}\right\|}^{\frac{-2}{b-1}}}{\overset{k}{\underset{s=1}{\sum }}{\left\|\chi_{i}-m_{s}\right\|}^{\frac{-2}{b-1}}}.$$ (3)

To solve Eqs. (2) and (3), the iterative method was adopted until the convergence condition was satisfied. The FCM algorithm steps are as follows. Given a clustering category c, iterative convergence conditions are set and each clustering center is initialized. Then, the following operation is repeated until the membership value of each sample is stable. First, according to Eq. (3), the current clustering center is used to calculate the membership function. Second, using the current membership function, the center of each cluster is recalculated according to Eq. (2).

**Fig. 2:**
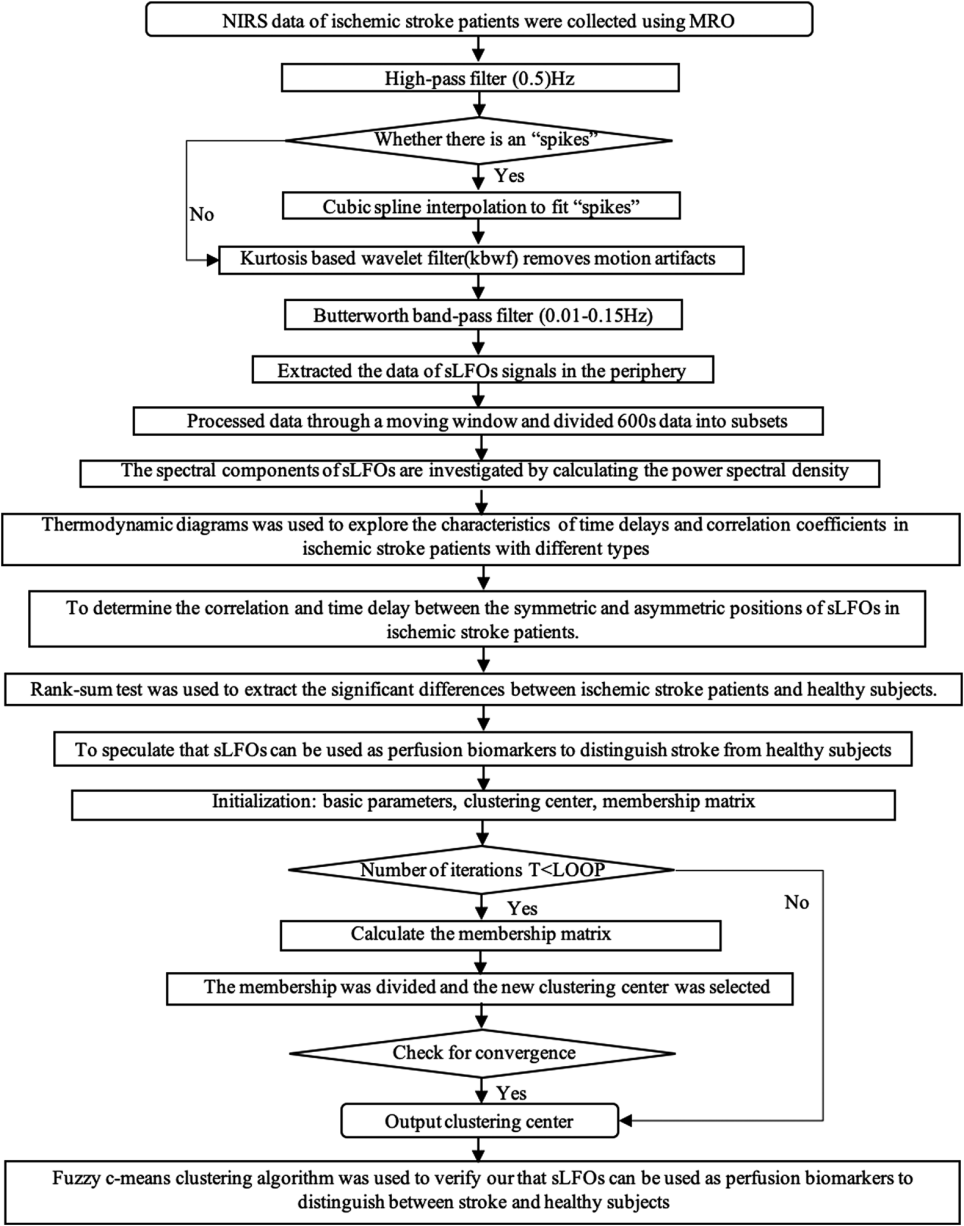
Flowchart of sLFO extraction and processing and data analysis methodology.

The clustering centers of each class and the membership values of each sample for each class are obtained when the algorithm converges, so as to complete the division of fuzzy clustering. The termination condition of the iteration is

$$\max_{ij}\{|\mu_{j}{\left(\chi_{i}\right)}^{\left(k+1\right)}-\mu_{j}{\left(\chi_{i}\right)}^{\left(k\right)}|\}<\varepsilon ,$$ (4)

where *k* is the number of iteration steps and ε is the error threshold. When the membership degree does not change significantly, it has reached a relatively optimal state (local optimal or global optimal), and the process converges to the local minimum or saddle point of target $J_{f}$. The flowchart of sLFO extraction and processing and data analysis methods is shown in Fig. 2.

## 3 Results

### 3.1 sLFOs Observed in Ischemic Stroke Patients

**Fig. 3:**
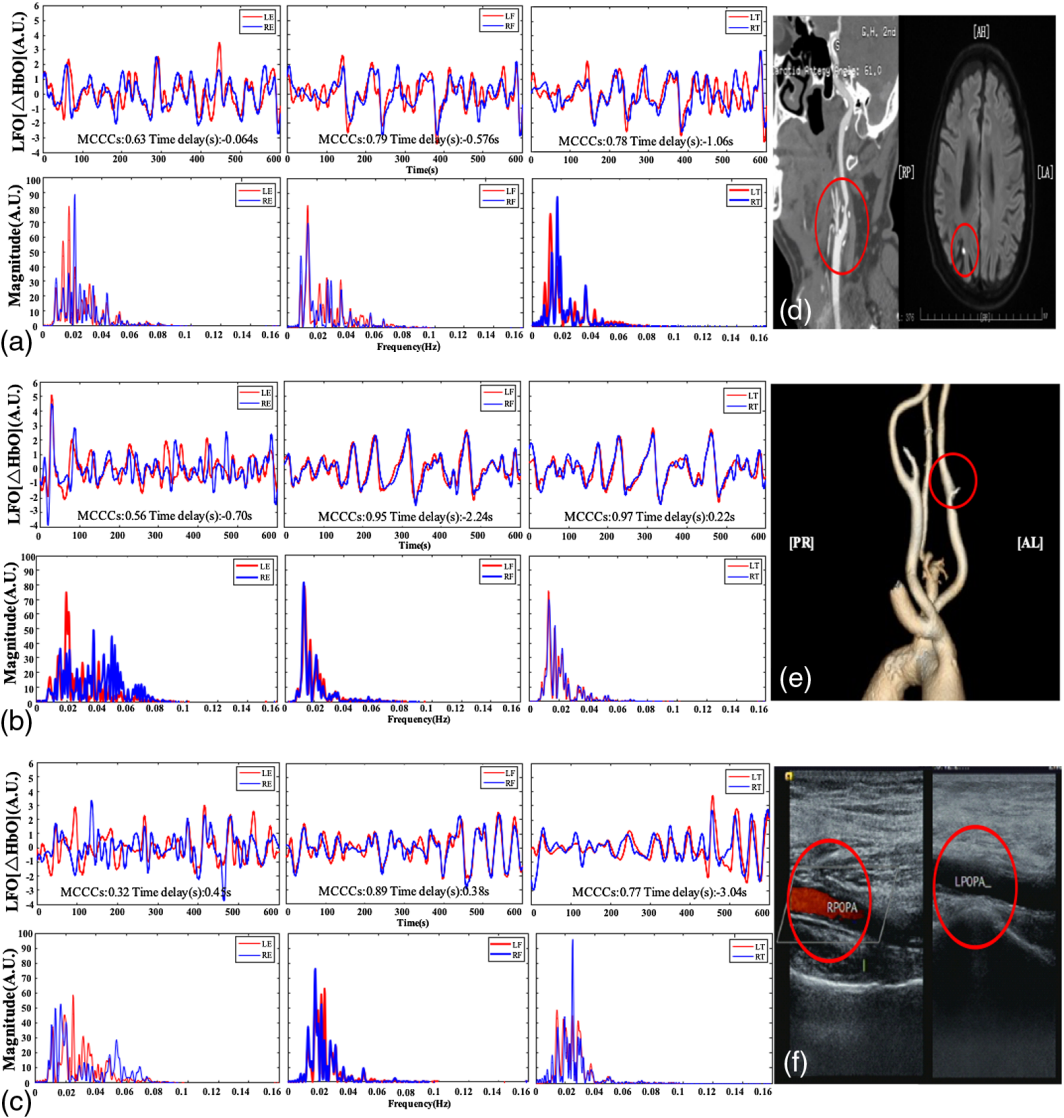
Time-domain waveforms, corresponding (a) sLFO PSD and (d) carotid artery CTA and brain MRI images of stroke patient (#441,265). Time-domain waveforms, (b) corresponding sLFO PSD and (e) carotid artery CTA image of stroke patient (#237,498). The time-domain waveforms, corresponding (c) sLFO PSD and (f) color Doppler ultrasonography images of left and right lower limb artery stenosis of stroke patient (#2,381,760).

We have the medical records of 27 ischemic stroke patients who had been diagnosed with ischemic stroke and had embolism. Among them, patient #441,265, patient #327,498, and patient #2,381,760 had CTA, MRI, and color ultrasound images providing embolization details. Patient #441,265 had been diagnosed with multiple vascular stenosis and cerebral infarction, but the conditions were not serious. The CTA and brain MRI images of the carotid artery from the patient with right internal and external carotid artery stenosis are shown in Fig. 3(d). The LE value ${\mathrm{SaO}}_{2}$ is 74.06%, and the RE value ${\mathrm{SaO}}_{2}$ is 74.28%. There is a slight difference in the standard value of 75% for venous oxygen. This indicates that the mild embolization did not cause a significant decrease in blood oxygen saturation. The pulse was 62.69 BPM in the left and right ears, which may indicate little difference between the degree of left and right carotid embolism. Similar results are also observed in sLFO data. The LE-RE correlation coefficient for sLFOs in Fig. 3(a) is 0.63. This is similar to the healthy subjects’ values (0.78±0.15), indicating that mild embolization did not alter the symmetry of the sLFO signal in ischemic stroke patients. Calculating the LE-RE time delay shows that the time delay (i.e., 1.15 s) exceeded the average value in healthy subjects. However, it was within the standard deviation (−0.12±1.47). This indicates that sLFOs are first detected in the RE and then in the LE. The further sLFO calculation results show that LF-RF (left fingertips and right fingertips) and LT-RT (left toes and right toes) also experienced varying delays. This may indicate that embolism’s effect on blood circulation in stroke patients is not only local but also global.

Patient # 327,498 was admitted with a diagnosis of left frontal lobe infarction. Figure 3(e) shows that this patient had left carotid artery stenosis. The ${\mathrm{SaO}}_{2}$ values in the LE and RE are 74.21% and 73.91%, respectively. The pulse is 71.29 BPM in the left ear and 71.99 BPM in the right ear. Finally, the sLFOs’ correlation coefficient between the two ears is 0.56, less than that of healthy subjects (0.78±0.15). Each of these differences indicates possible asymmetric effects of the stroke in both ears.

The ischemic stroke patient (#2,381,760) in Fig. 3(c) has peripheral vascular disease. The LT and RT blood oxygen contents are 73.61% and 73.64%, respectively, lower than the 75% standard level for healthy subjects. This suggests that the embolism affects the blood supply and reduces oxygen levels in the legs. The time delay between LT and RT is −3.04  s, different from that of healthy subjects (0.68±1.17). This indicates that there was asymmetric perfusion in both branches (to LT and to RT). We report these signals’ power spectral density in Fig. 3 and calculate the PSD of LE, RE, fingers, and toes. The results show that the signals with high correlation coefficients have better overlapping PSD. In addition, these spectrum components are all within the 0 to 0.1 Hz range. Moreover, the earlobe’s spectrum width is generally higher than that of the fingertips and toes.

### 3.2 Correlation Coefficients and Time Delays of sLFOs in Peripheral Positions

**Fig. 4:**
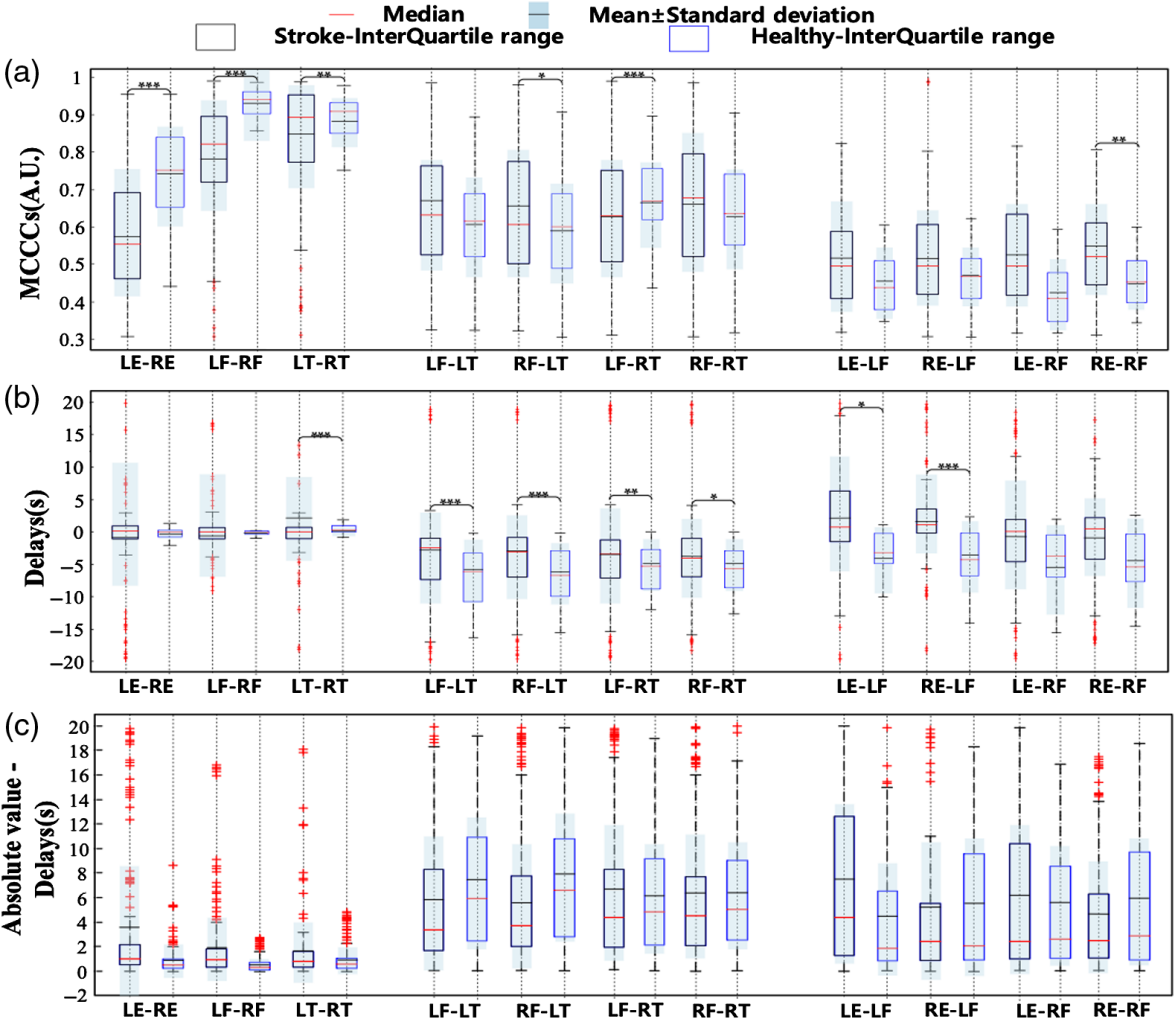
Statistical results of (a) correlation coefficients, (b) time delays, and (c) absolute value–time delays variance across stroke patients and healthy subjects showing peripheral sources (blue-filled and blue-void boxplots for stroke and healthy signals, respectively) in symmetric and asymmetric positions for a paired-sample rank-sum test P<0.001 (***), P<0.01 (**), P<0.05 (*).

As shown in Fig. 4(a), most correlation coefficients for LE–RE, LF–RF, and LT–RT in ischemic stroke patients is distributed between 0.45 to 0.68, 0.7 to 0.9, and 0.75 to 0.95, respectively. The baseline values of correlation coefficients and time delays in healthy subjects have been established in our previous studies.^18^ We used a rank-sum test to extract the significant differences between ischemic stroke patients and healthy subjects. To reduce the error rate for positive results (the FDR value), we used the Benjamini–Hochberg method to correct the results, keeping the false-positive probability below 0.05. The P-values, before and after correction, are shown in Table 1 (Appendix A).

**Fig. 5:**
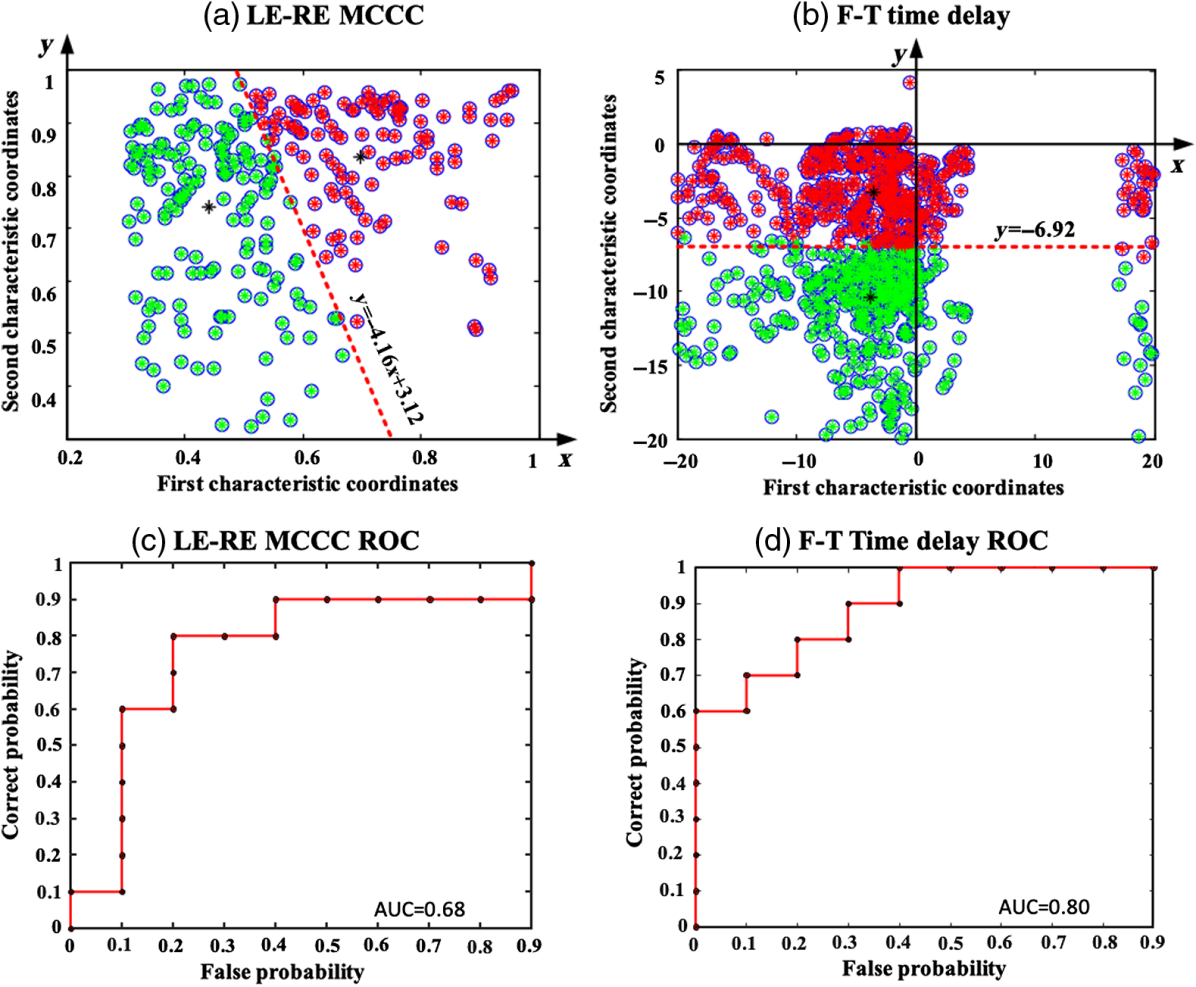
(a) LE-RE MCCC classification and (b) F-T time delay classification between ischemic stroke patients and healthy subjects using the FCM clustering algorithm. ROC curve for (c) LE-RE MCCC classification and (d) F-T time delays.

In Fig. 4, the significant differences between the results of ischemic stroke patients and healthy subjects in LE-RE and LF-RF (p<0.001) are greater than those in LT-RT (p<0.01). The F-T correlation coefficients in ischemic stroke patients are concentrated from 0.5 to 0.8, and significant differences occur for RF-LT (p<0.05) and LF-RT (p<0.001). The E-F correlation coefficients in ischemic stroke patients are concentrated around 0.4 to 0.65; significant differences occur for RE-RF (p<0.01) compared with healthy subjects that concentrate at 0.35 to 0.5. As shown in Fig. 4(b), the time delays of symmetrical positions (LE-RE, LF-RF, and LT-RT) in ischemic stroke patients is significantly greater than that in healthy subjects with almost zero time delays (p<0.001). There are positive and negative differences in the time delays in asymmetric positions (F-T and E-F) between ischemic stroke patients and healthy subjects, i.e., the delays in healthy subjects in asymmetric positions are concentrated below 0, while some ischemic stroke subjects’ data are positive. The highly significant differences of time delays are for F-T [LF-LT (P<0.001), RF-LT (P<0.001), LF-RT (P<0.01), and RF-RT (P<0.05)]. We needed to eliminate the influence of the positive and negative time delay values on the analysis results. Therefore, the absolute values of the time delays between the symmetrical and asymmetric positions for ischemic stroke are taken, and the significant differences are derived again, as shown in Fig. 5(c). There is no significant difference between the absolute values of ischemic stroke patients’ time delays and those of healthy subjects. We also find no linear relationships between the sLFOs’ correlation coefficients and time delays. This may be related to the disruption of sLFO transmission in the body by embolization. We also calculated the pulse rates of 25 stroke patients and 25 healthy subjects, as shown in Fig. 8 (Appendix B).

### 3.3 sLFO Fuzzy Clustering Between Ischemic Stroke Patients and Healthy Subjects

We extracted the characteristics of the differences between the LE-RE MCCCs and the F-T time delays between healthy subjects and ischemic stroke patients (see Sec. 3.2). We used the fuzzy *c*-means clustering algorithm to cluster these characteristics with significant differences, as shown in Fig. 5. In Fig. 5(a), the LE-RE correlation coefficients are divided into two categories along the equation “y=−4.16x+3.12.” In Fig. 5(b), the F-T time delays are divided into two categories along the equation “y=−6.92.” In addition, we calculated an ROC curve to indicate the classification algorithm’s sensitivity and specificity. The ROC curve for the fuzzy classification results is shown in Figs. 5(c) and 5(d), and the results show that ${\mathrm{AUC}}_{\mathrm{LE}\text{-}\mathrm{RE}\;\mathrm{MCCC}}$ is 0.68 and ${\mathrm{AUC}}_{F\text{-}T\text{ Time delay}}$ is 0.80. These results validated our hypothesis that sLFOs’ signal can serve as perfusion biomarkers to differentiate ischemic stroke patients from healthy subjects. In our experiments, we not only conducted two-category analysis but also calculated the multiclassification results, as shown in Fig. 9 (Appendix C).

## 4 Discussion

### 4.1 Spectrum Characteristics of sLFOs

**Fig. 6:**
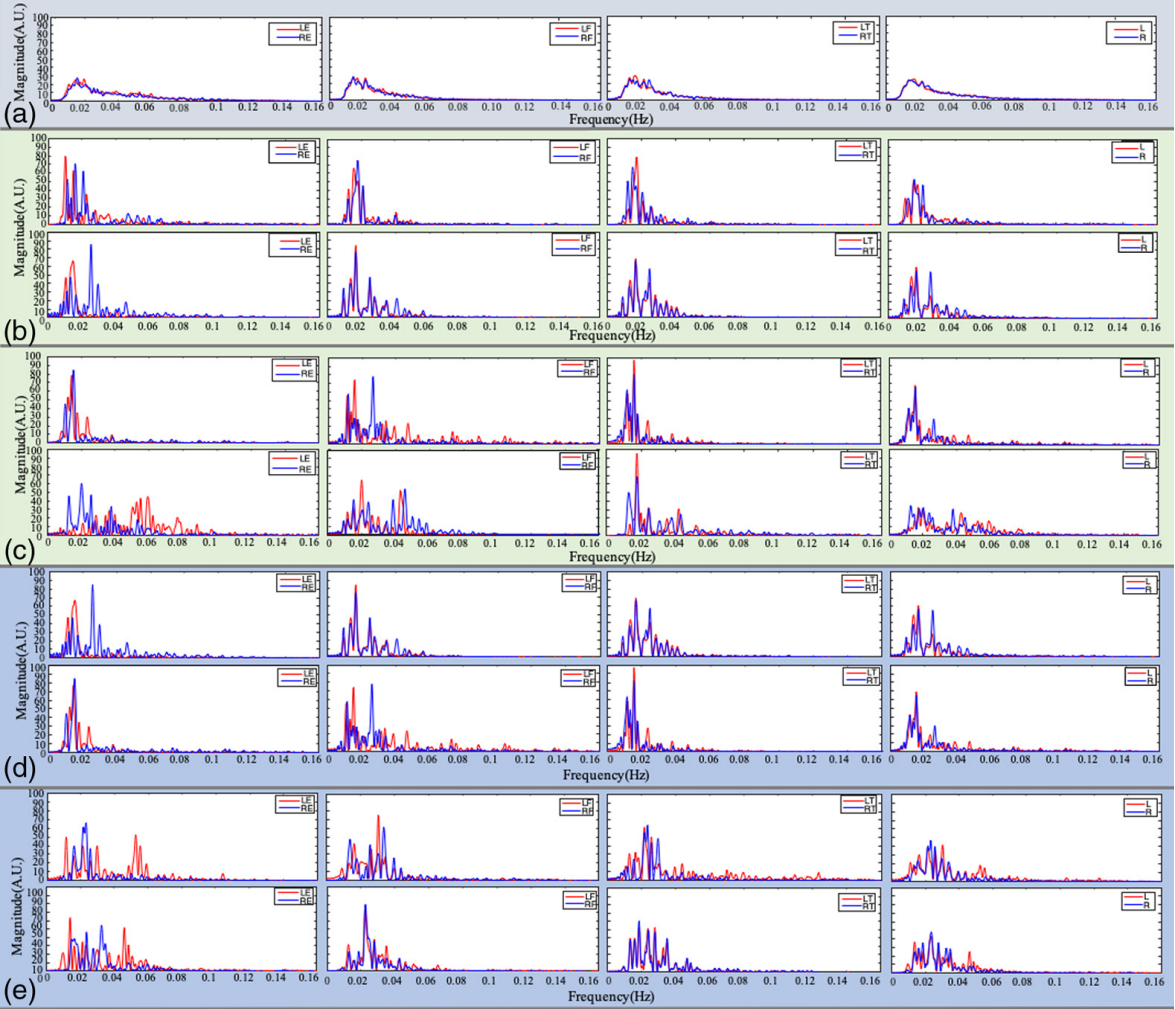
(a) Average power spectral density of all ischemic stroke patients; power spectral density and the average values for both sides of ischemic stroke patients with (b) bilateral internal carotid artery stenosis, (c) external carotid artery stenosis, (d) lower extremity artery stenosis, and (e) severe limb paralysis.

The sLFO power spectrum density analysis reveals relationships between energy variation and frequency. Our previous study^18^ explored the spectrum characteristics in the periphery of healthy subjects in resting states. The results showed that the spectral content in LE-RE focused on 0 to 0.12 Hz; in LF-RF and LT-RT, it focused on 0 to 0.08 Hz (the peak appeared near 0.02 Hz).^18^ This study explores sLFOs’ PSD characteristics in the peripheries of ischemic stroke patients. For the study to be comprehensive, we explored ischemic stroke patients’ PSD with bilateral internal carotid artery stenosis, external carotid artery stenosis, lower extremity artery stenosis, and severe limb paralysis, as shown in Fig. 6. We found that the signals from symmetrical positions of ischemic stroke patients have small overlapping PSD, especially in the earlobes. This indicated that the embolism breaks the blood transmission symmetry, disrupting the blood circulation balance on both sides of the body. The earlobes’ power spectral density ranges from 0 to 0.1 Hz, generally higher than that of fingertips and toes (0 to 0.08 Hz). The symmetrical earlobes’ power spectral density’s similarity is generally lower than that of fingertips and toes. This indicates that earlobes may contain more physiological information. To generalize the findings, we calculated the power spectral density of all ischemic stroke patients and obtained the average, as shown in Fig. 6(a). The width of the average LE-RE power spectrum density remained within 0 to 0.01 Hz, still higher than that of LF-RF and LT-RT at 0 to 0.08 Hz. In addition, there was no significant difference in spectral components between different types of ischemic stroke patients. This demonstrates the consistency of the spectral components.

### 4.2 Time Delays and Correlation Coefficients

**Fig. 7:**
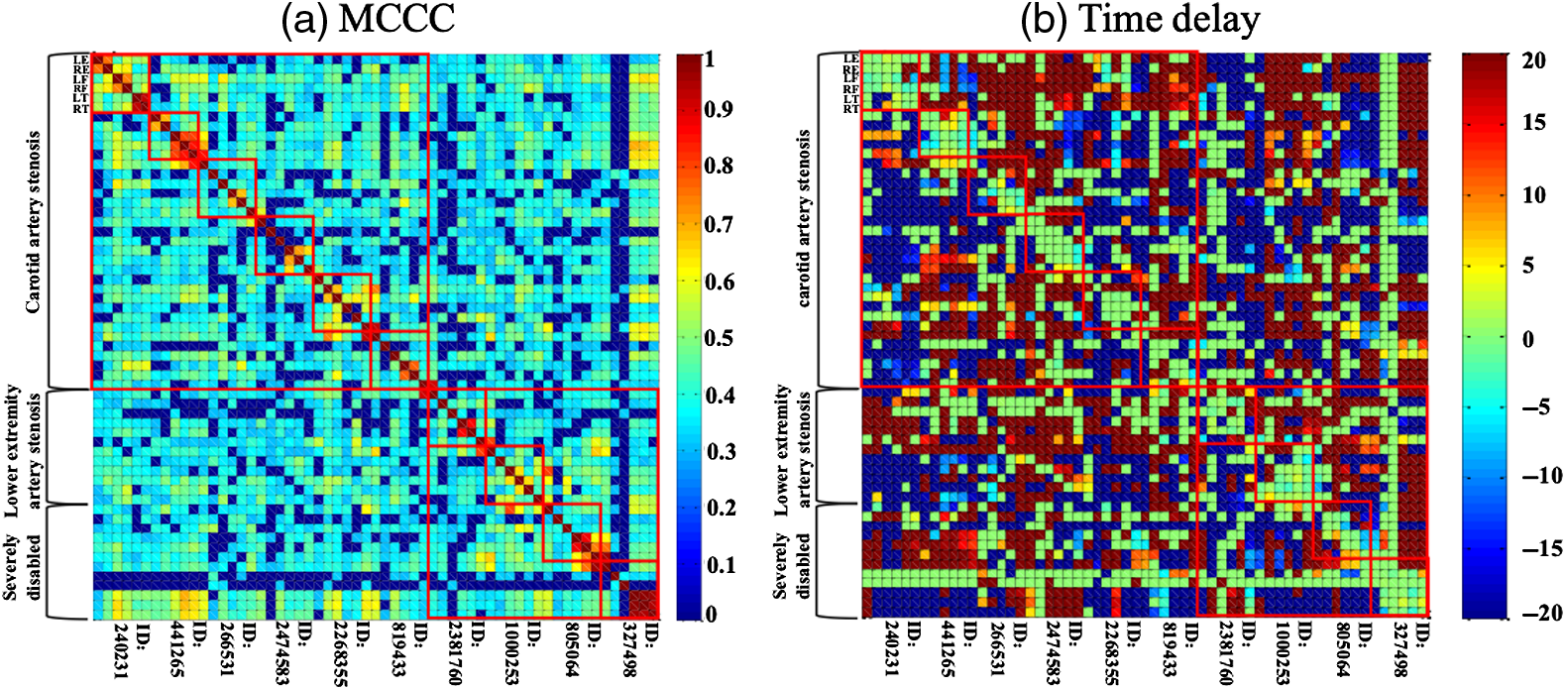
Statistical results for (a) MCCC delays and (b) cross-carotid artery stenosis, lower extremity artery stenosis, and severe disability.

Exploring the time delays and correlation coefficients of peripheral sLFOs may reveal the underlying coupling mechanism. From the perspective of vascular distance from the cardiovascular system, the times of blood arrival at the earlobes, fingertips, and toes should theoretically increase. However, in Fig. 7(b), the E-F time delays in ischemic stroke patients are positive. This indicates that blood flows take longer to arrive at the earlobes than at the fingertips. This is because the arteriosclerosis may have contributed to the interruption or retardation of the blood flow. The same results can be observed in the time delays for F-T. In Fig. 7(a), the correlation coefficients for the ears and fingertips of ischemic stroke patients are higher than those of healthy subjects (p<0.05). This is inconsistent with the physiological structure of the human body and probably means that the blood flow rules have been disturbed. The embolism in ischemic stroke patients may adhere to the wall of a blood vessel or it may flow with the blood. Blood flow can be blocked by thrombus, arteriosclerosis, or a combination of both, leading to unpredictable correlation and delays.

Before discussing the physiological meaning, we would like to address the possibility that these results are the result of spurious correlations. Bandpass filtering and selecting the maximum cross correlation over a time range can inflate the correlation results’ significance.^32^ Figure 3 shows that the sLFOs have complex, inharmonic frequency structure, meaning that the sLFO signals are aperiodic in most cases; they have no “phase,” only time delay. The maximum cross correlation values are greater than the neighboring negative/positive values (p<0.05). Therefore, accurate corresponding time delays can be extracted. In our previous study,^32^ random time series were filtered (<0.1  Hz) and cross correlated to assess the effect on the p-value. The findings showed that MCCC must be >0.28 to achieve a p-value below 5%. Therefore, in this study, we selected a 0.3 threshold for MCCC. According to the range of global circulation times^29^^,^^33^ (10 to 18 s, with an average of 14.7 s), the search range for time delays is restricted to ±20  s in this study.

In previous work,^18^ sLFOs were thought to originate in the cardiovascular system and flow with the blood throughout the body. Therefore, symmetry propagation is the characteristic of sLFOs in healthy subjects. However, vascular lesions disrupt the characteristics of symmetrical transmission. The relative delay and amplitude of the various LFO frequency components will be modified, depending on the specific vascular path it takes. We expect to find diminished correlation coefficients in ischemic stroke patients’ symmetrical positions, and the degree of reduction is related to the patient’s condition. This is confirmed in Fig. 7, which offers the most salient support of our assumption. Moreover, we also expect that sLFOs in the ischemic stroke patients’ asymmetric positions do not follow the rule of reaching first the earlobes, then the fingers, and finally the toes. This also can be seen in Fig. 7(b). Finally, we found that each patient’s time delays and correlation coefficients in symmetric and asymmetric positions vary. This may be related to several factors. For example, sLFOs “move” with the blood throughout the body. Their transmission speed is correlated with blood viscosity, blood pressure, and pathology. A patient’s blood vessel disease affects their blood flow. In turn, this affects the sLFOs signals’ transmission characteristics [such as maximum cross correlation coefficients (MCCCs) and time delays]. These effects may either enhance or attenuate the correlation coefficients and time delays of the (a)symmetrical positions in the periphery. However, this does not mean that sLFOs change speed depending on the pathology. Many factors affect correlation coefficients and time delays, such as scanning environment and temperature. This experiment was conducted in a more relaxed environment than the FMRI study. Thus, the scanning environment likely had less influence. Furthermore, the experiment was conducted at a comfortable temperature. This reduced the influence of temperature. Moreover, our previous work^18^ has found no significant correlation between age and time delay.

### 4.3 Importance of Stroke Patients’ Ear Signals

According to the literature,^34^ the ear blood vessels contain important branches of the external carotid artery supplying blood to the scalp and ears. In other words, signals from the earlobes may carry information about the carotid artery, scalp, and face. This point can be observed in Figs. 4 and 6. In Fig. 4, sLFOs in the LE and RE show a wider power spectrum component. In Fig. 6, the earlobe position signal is more sensitive than that of hands and feet. These results indicate that the LE and RE can be important measurement points and may potentially contain more physiological information.

Each type of tissue has different optical properties—the optical parameters are locally different. The earlobes have no bone and little fat and are simpler and more uniform than other peripheral locations. Thus, the above characteristics of earlobes are suitable for the application of the Beer–Lambert law. The earlobes are thinner than other peripheral tissues, and thus conducive to photon transmission events. A previous experiment has evaluated the contributions of ICA and ECA to changes in the concentration of oxyhemoglobin (HbO) and deoxygenated hemoglobin (Hb) in carotid endarterectomy by NIRS.^32^ The study found that both the ECA and ICA vascular territories contribute to NIRS changes. The primary blood supply to the earlobes is from the external carotid artery. In addition, the signals in the earlobes may reflect fluctuations in blood flow to the carotid artery. Therefore, patients’ ear sLFO signals play an important role in stroke research.

### 4.4 Limitations of this Study

The number of subjects in this study was fairly low. Due to stroke patients’ physical limitations, we were only able to conduct the resting state experiment in this study. Further studies could explore the characteristics of sLFOs in ischemic stroke patients in task states (e.g., paced breathing and passive leg lifting).

## 5 Appendix A

We improved the judgment standard (P-value) to reduce the positive results’ error rates (FDR value). Then, we used the Benjamini–Hochberg method to correct the results. P-values before and after correction are shown in Table 1; the correction can maintain a false-positive probability below 0.05.

**Table 1:** *P*-values before and after correction for multiple comparisons.

Positions	MCCC		Time delay	
	*P*-value	Adjusted *P*-value	*P*-value	Adjusted *P*-value
LE-RE	5.39e-31	2.96e-30	0.130	0.179
LF-RF	1.63e-32	1.79e-31	0.625	0.625
LT-RT	0.003	0.006	6.94e-13	7.64e-12
LF-LT	0.107	0.168	2.71e-8	9.93e-08
RF-LT	0.027	0.050	1.85e-08	1.02e-07
LF-RT	1.70e-5	6.23e-5	0.002	0.005
RF-RT	0.169	0.233	0.002	0.003
LE-LF	0.513	0.564	0.031	0.048
RE-LF	0.562	0.562	3.84e-6	1.06e-05
LE-RF	0.414	0.506	0.557	0.613
RE-RF	0.002	0.006	0.167	0.204

## 6 Appendix B

**Fig. 8:**
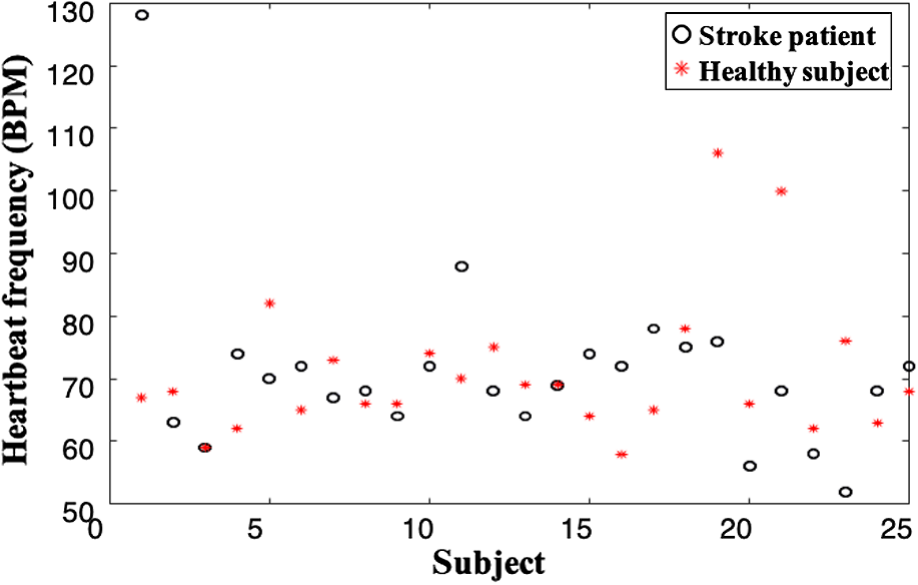
Heart rate comparison between 25 ischemic stroke patients and 25 healthy subjects.

We compared the heart rate differences of 25 ischemic stroke patients with 25 healthy subjects. The results showed no significant differences in heart rate between ischemic stroke patients and healthy subjects, as shown in Fig. 8.

## 7 Appendix C

**Fig. 9:**
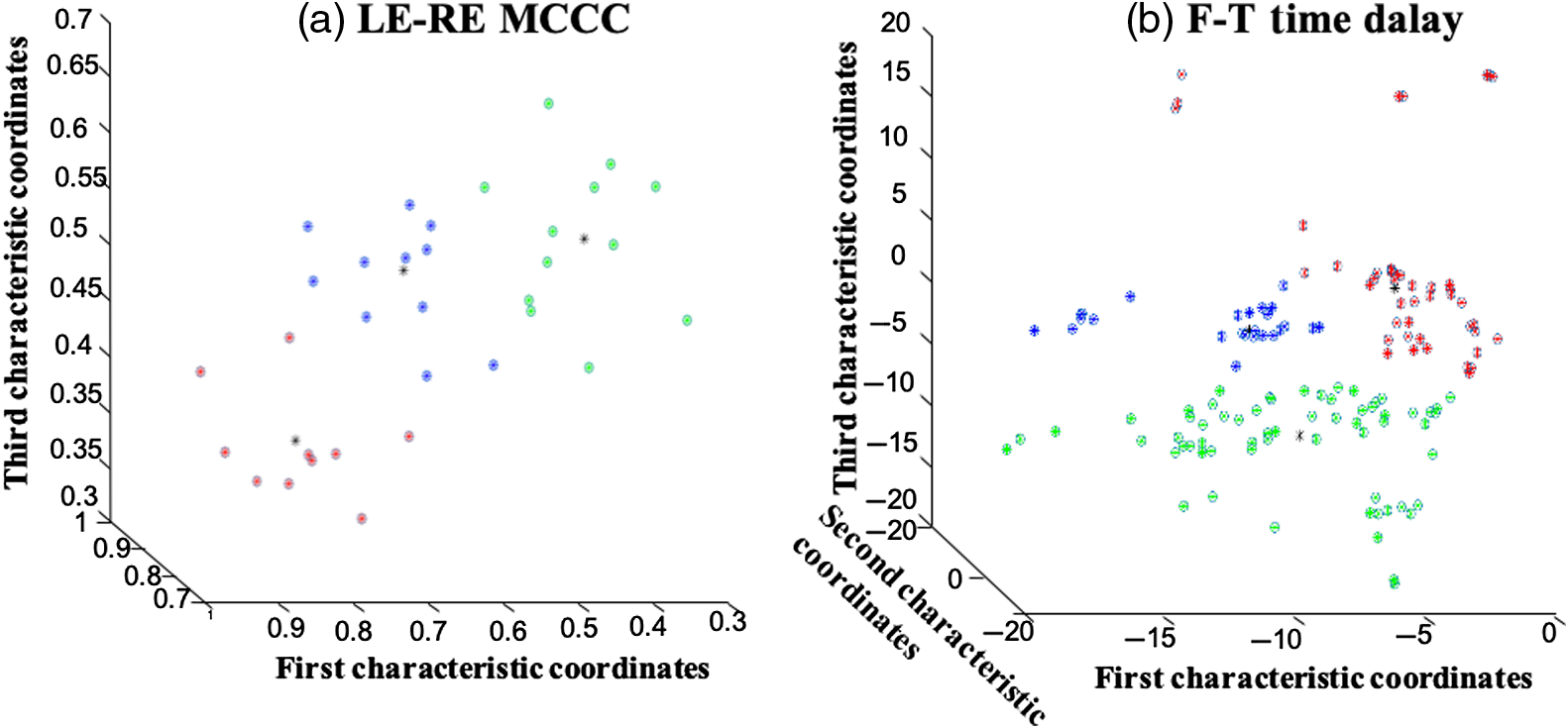
(a) LE-RE MCCC classification and (b) F-T time delay classification between healthy subjects, carotid stenosis, and peripheral vascular disease using the FCM clustering algorithm.

We used a fuzzy c-means clustering algorithm to divide the data into healthy subjects, those with carotid stenosis, and those with peripheral vascular disease. This is shown in Fig. 9.
